# Cell binding, uptake, and infection of influenza A virus using recombinant antibody-based receptors

**DOI:** 10.1128/jvi.02275-24

**Published:** 2025-04-10

**Authors:** Oluwafemi F. Adu, Milagros Sempere Borau, Simon P. Früh, Umut Karakus, Wendy S. Weichert, Brian R. Wasik, Silke Stertz, Colin R. Parrish

**Affiliations:** 1 Department of Microbiology and Immunology, College of Veterinary Medicine, Baker Institute for Animal Health, Cornell University 214915, Ithaca, New York, USA; 2 Institute of Medical Virology, University of Zurich 27217, Zürich, Switzerland; 3 Department of Veterinary Sciences, Ludwig-Maximilians-University 9183, Munich, Germany; University Medical Center Freiburg, Freiburg, Germany

**Keywords:** orthomyxovirus, host-cell interactions, receptor binding, experimental tools, endocytosis

## Abstract

Human and avian influenza A viruses bind to sialic acid (Sia) receptors on cells as their primary receptors, and this results in endocytic uptake of the virus. While the role of Sia on glycoproteins and/or glycolipids for virus entry is crucial, the roles of the carrier proteins are still not well understood. Furthermore, it is still unclear how receptor binding leads to infection, including whether the receptor plays a structural or other roles beyond being a simple tether. To enable the investigation of the receptor binding and cell entry processes in a more controlled manner, we have designed a protein receptor for pandemic H1 influenza A viruses. The engineered receptor possesses the binding domains of an anti-HA antibody prepared as a single-chain variable fragment (scFv) fused with the stalk, transmembrane, and cytoplasmic sequences of the feline transferrin receptor type-1 (fTfR). When expressed in cells that lack efficient display of Sia due to a knockout of the *Slc35A1* gene, which encodes for the solute carrier family 35 transporter (SLC35A1), the anti-H1 receptor was displayed on the cell surface, bound virus, or hemagglutinin proteins, and the virus was efficiently endocytosed into the cells. Infection occurred at similar levels to those seen after reintroducing Sia expression, and lower affinity receptor mutants displayed enhanced infections. Treatment with clathrin-mediated endocytosis (CME) inhibitors significantly reduced viral entry, indicating that virus rescue by the antibody-based receptor follows a similar internalization route as Sia-expressing cells.

**IMPORTANCE:**

Influenza A viruses primarily circulate among avian reservoir hosts but can also jump species, causing outbreaks in mammals, including humans. A key interaction of the viruses is with host cell sialic acids, which vary in chemical form, in their linkages within the oligosaccharide, and in their display on various surface glycoproteins or glycolipids with differing properties. Here, we report a new method for examining the processes of receptor binding and uptake into cells during influenza A virus infection, by use of an engineered HA-binding membrane glycoprotein, where antibody variable domains are used to bind the virus, and the transferrin receptor uptake structures mediate efficient entry. This will allow us to test and manipulate the processes of cell binding, entry, and infection.

## INTRODUCTION

Influenza A viruses (IAVs) are negative-sense single-stranded segmented RNA viruses of the *Orthomyxoviridae* family. They have an envelope that anchors two key glycoproteins, hemagglutinin (HA) and neuraminidase (NA), which mediate infection and tropism of the virus (1–3). For most IAVs, HA facilitates entry into competent cells by binding to sialic acids (Sia), often in the form of N-acetylneuraminic acid (Neu5Ac) as the terminal glycan of an oligosaccharide linked to either surface glycoproteins or glycolipids (4–6). Bat IAVs (H17 and H18) and avian H19 are an exception to Sia-mediated entry, as those use major histcompatibility complex (MHC) class II glycoproteins for attachment and uptake (1, 7–9).

Host-specific evolution of IAV-HA leads to observed selective binding of differently linked Sias. Human IAVs favor Sia with an α2,6 linkage to a neighboring galactose or GalNac residue, while avian IAV strains favor α2,3-linked Sia (10–12). Additionally, the NA plays a significant role in facilitating IAV infection due to its ability to cleave Sia, thereby reducing virus clustering during viral budding and release. NA also modifies non-productive HA–Sia interactions with mucus within the respiratory tract (2, 13). This, in turn, promotes productive viral attachment and infection of the underlying epithelial cells. Furthermore, NA has been shown to sometimes also mediate Sia binding and to also directly interact with the immunomodulatory cell adhesion molecule CEACAM6 (CD66c) to promote IAV entry into competent cells (14). However, whether this CD66c interaction is independent of Sia is unknown. In total, for most virus:host combinations, there is holistic HA–NA–Sia interaction balance that is necessary to promote IAV fitness (2).

Following Sia binding, viral entry involves significant levels of clathrin-mediated endocytosis (CME) (15–17). However, it is still unclear how Sia binding efficiently leads to entry through that pathway, and also whether it involves the preferential use of Sia expressed on specific surface glycoproteins or glycolipids (4, 18, 19). It is theorized that attachment to Sia alone may not be sufficient to trigger signaling cascades necessary for effective internalization, and also that efficient endocytosis involves binding of multiple receptors by the multivalent influenza virus particle, which also increases the avidity of binding (20).

In addition to Sia, multiple receptors or co-receptors have been suggested for IAVs. These include nucleolin (21, 22), the calcium voltage pump CaV1.2 (23–25), NK cell-specific NKp44/46 (26, 27), and the metabotropic glutamate receptor subtype 2 (28). These receptors have been suggested to aid in virus attachment by directly interacting with the HA. Other receptor candidates or facilitating molecules include members of the receptor tyrosine kinases such as the epithelial growth factor receptor (20) or the G protein-coupled receptors and associated proteins like β-arrestin and the free fatty acid receptor 2 (29, 30). Those are thought to engage (likely indirectly) with the virus upon uptake.

Alternative pathways for IAV entry may also involve the interaction of N-linked glycans present on the viral HA or NA with various C-type lectins expressed on many immune cells. These include DC-SIGN on dendritic cells (31), macrophage mannose receptor and macrophage galactose-type lectin on macrophages (32), and Langerin on Langerhans cells in the human airway (33). These interactions may play direct or indirect roles in IAV uptake and internalization, leading to infection.

Understanding the specific interactions between IAV and the many above-described candidate receptor components and their roles in directing viral entry and infection has been challenging. Several factors contribute to this difficulty, including the presence of different IAV strains that may engage distinct co-receptors, diverse tropisms for various cell types which may involve different host cell receptors or co-receptors, possible redundancy in the entry mechanisms of the virus, and difficulties associated with analyzing and altering the glycan-protein expression in a predictable way. In addition, methods currently employed may lack specificity and sensitivity, and *in vitro* systems may not fully recapitulate the *in vivo* processes in the respiratory or gastrointestinal tracts of the natural hosts.

To provide new controllable methods for studying IAV entry and uptake mechanisms, we have developed a novel antibody-based receptor chimera that mediates pandemic H1N1 (pH1N1) entry/infection without the involvement of Sia. This allows for more flexibility and manipulation of the binding, entry, and infection processes in different systems and cell types. We show that a chimeric glycoprotein receptor rescued pH1N1 entry and infection in both the human embryonic kidney 293 (HEK 293) and the adenocarcinoma human alveolar-derived epithelial cells (A549) that lacked cell surface Sia expression.

## MATERIALS AND METHODS

### Cells and viruses

HEK 293 (HEK) and A549 cells were obtained from the American Type Culture Collection and were cultured at 37°C, 5% CO_2_. HEK *Slc35A1* knockout (KO) cells were prepared as previously described in reference 5. A549 *Slc35A1* KO cells were generated by CRISPR-Cas9-mediated genome editing. Briefly, A549 cells were reverse-transfected with pre-assembled ribonucleoproteins consisting of Alt-R S.p. Cas9 nuclease (IDT) in complex with Alt-R CRISPR-Cas9 crRNA that targets exon 1 of the *Slc35A1* gene (*Slc35A1*_crRNA: [AltR1] rGrA rCrCrC rArGrU rUrCrU rCrArC rCrUrC rUrCrG rGrUrU rUrUrA rGrArG rCrUrA rUrGrC rU [AltR2] [IDT]) and Alt-R CRISPR-Cas9 tracrRNA – ATTOTM 550 (IDT), using RNAiMax (Thermo Fisher Scientific). At 48 h post-transfection, single-cell clones were generated by limiting dilution in 96-well plates and screened by next-generation sequencing (NGS). Genomic DNA was extracted from single-cell clones using QuickExtract DNA Extraction Solution 1.0 (Lucigen), and the region of interest was amplified by two consecutive PCRs. The first PCR was run with primers containing adapters for TruSeq HT index primers (indicated by underlined nucleotides): SLC35A1_frw: 5′-CTT TCC CTA CAC GAC GCT CTT CCG ATC TTC TAT GAC CAC AAG GGG CGG TC-3′ and SLC35A1_rev: 5′-GAC TGG AGT TCA GAC GTG TGC TCT TCC GAT CTA GCG GCT CCA CGC AAA CTC C-3′. The second PCR was run using TruSeq HT index primers D50x and D70y to generate barcoded amplicons. After gel extraction, barcoded amplicons were analyzed by MiSeq (Illumina). A genotypically *Slc35A1* KO clone was selected and phenotypically validated by *Sambucus nigra* lectin (SNA) and *Maackia amurensis* II staining (see below for method). All cells used in this study were grown and maintained in Dulbecco’s modified Eagle medium (DMEM, Gibco) with 10% fetal calf serum and 50 µg/mL gentamicin (HEK) or 100 U/mL penicillin and 100 µg/mL streptomycin (A549) (Gibco).

A/California/04/2009 (Ca’09) was derived from reverse genetics plasmids as previously described (34). A/Netherlands/602/2009 and Neth/09-*Renilla* were generated following previously described protocols (35).

### Lectin staining

#### Flow cytometry analysis

HEK cells were grown to sub-confluency in a 12-well dish, collected using Accutase (Sigma), and blocked with Carbo-Free Blocking Solution (Vector Laboratories). Cells were incubated with fluorescein (FITC)-conjugated SNA or MAL I (Vector Laboratories) for 30 min on ice, and signal intensities for at least 10,000 live cells were assessed using a Millipore Guava EasyCyte Plus flow cytometer (EMD Millipore). Data were analyzed with FlowJo software (TreeStar).

For A549 cells, the cells were washed with phosphate-buffered saline (PBS), detached with 0.25% trypsin-EDTA (Thermo Fisher Scientific), resuspended in fluorescence-activated cell sorting (FACS) buffer (PBS with 2% bovine serum albumin (BSA) and 1 mM EDTA), and incubated with biotinylated SNA or MAL II lectins (Vector Laboratories) for 1 h at 4°C. After washing, cells were stained with allophycocyanin (APC)-streptavidin (BioLegend) and LIVE/DEAD Fixable Near-IR Dead Cell Stain (Thermo Fisher Scientific) for 30 min at 4°C. APC signal intensities of at least 5,000 live cells were analyzed using a BD FACSVerse flow cytometer with BD FACSuite software. Data were analyzed in FlowJo. For both cell types, background signal intensities were subtracted, and values were normalized to their wild-type versions using GraphPad Prism.

#### Immunofluorescence analysis of lectin binding

HEK cells were seeded into poly-D-lysine-treated glass slides (Invitrogen), fixed with 4% paraformaldehyde, blocked with Carbo-Free Blocking Solution (Vector Laboratories), and stained with FITC-conjugated SNA or MAL I lectins I (Vector Laboratories) and 4′,6-diamidin-2-phenylindol (DAPI). Images were acquired using a Zeiss AxioSkop HBO 50 fluorescence microscope.

For A549 cells, 120,000 cells per well were seeded onto glass coverslips and incubated for 24 h at 37°C. Cells were washed with PBS, fixed with 4% paraformaldehyde (PFA), blocked with PBS containing 2% BSA, and stained with biotinylated SNA or MAL II lectins. Samples were then stained with Alexa Fluor 647-streptavidin (Invitrogen) and DAPI (Sigma-Aldrich), mounted using ProLong Gold Antifade Mountant (Thermo Fisher Scientific), and imaged using a Leica SP8 confocal laser scanning microscope with a 63× objective. Images were acquired with LAS X software and processed with LAS AF Lite.

### Construction of chimeric receptors and HA-human Fc probe

**Fig 1:**
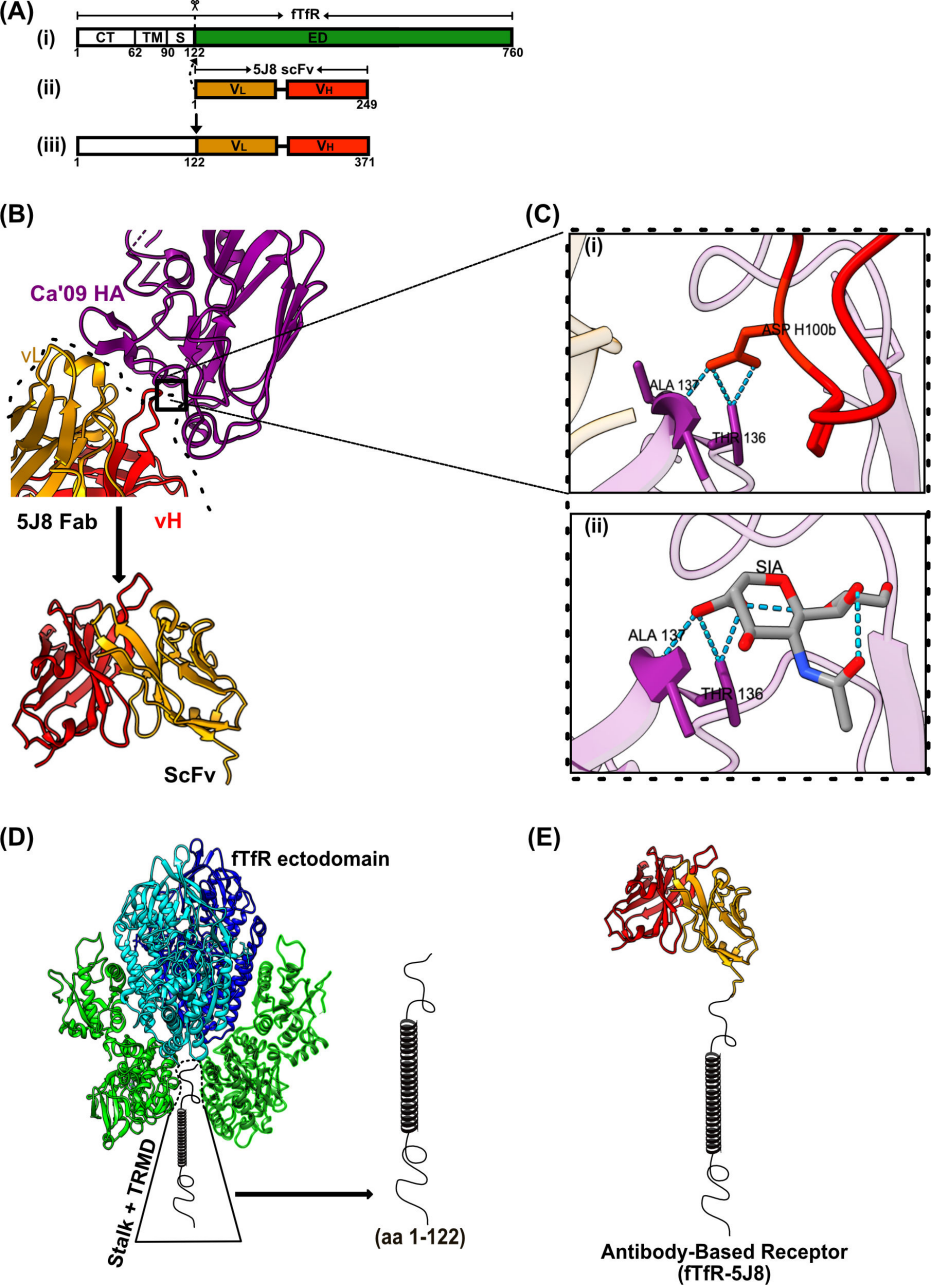
Design of antibody-based IAV receptor. (**A**) Cartoon showing the construction and cloning of the fTfR-5J8 chimera. (i, ii) Heavy and light variable chains of MAb 5J8 were cloned as scFv constructs in-frame onto residues 1–122 of the fTfR (iii) construct. (**B**) Crystal structure of Fab 5J8: A/Ca/07/2009-HA1 (PDB: 4M5Z). Purple (HA), gold (Fab vL), and red (Fab vH). (C) Magnified H-bond interactions of HCDR3 (red) Asp 100 interactions with (i) Ala 137 and Thr 136 HA residues. (ii) Identical H-bond interactions of Sia with Ala 137 and Thr 136 HA residues (PDB:3UBE) (36). (D) Cartoon of the TfR structure showing the ectodomain with transmembrane (TRMD) and stalk domains (aa 1–122). (E) Cartoon of antibody-based receptor monomer (scFv from Fab 5J8) fused to the stalk and transmembrane and cytoplasmic domains of the fTfR). Colors in the fTfR are similar to the different domains as defined for the human TfR; green (bottom half), protease-like domain; green (top half), apical domain; cyan and blue, helical domain (37).

Structures of the Fab 5J8 complexed with IAV-HA and details of their interactions were obtained from the Protein Data Bank (PDB: 4M5Z) (36). The chimeric antibody-based receptor (fTfR-5J8) was prepared from the fTfR cytoplasmic, transmembrane, and stalk domains (residues 1–122) (37) linked to the human monoclonal antibody (MAb) 5J8 (36, 38) single-chain variable fragments (scFvs). The fTfR-5J8 receptor was prepared by in-frame cloning of the heavy- and light-chain variable sequences linked by poly-GS linker sequences (Fig. 1A through E) and expression from the pcDNA3.1 (−) vector under the control of the cytomegalovirus (CMV) immediate early promoter (39). The intact fTfR in the same expression vector was also used as control (37, 40).

To produce a probe for HA binding, the H1N1 Ca’09 HA ectodomain was fused via its C-terminus in-frame to the human IgG1 Fc, and the Gp64 baculovirus secretion peptide fused to the N-terminus as described previously (41, 42). Genes were synthesized and cloned into pFASTBac-1 (Life Technologies) to generate recombinant bacmids according to the manufacturer’s protocol and as described previously (42). Recovered recombinant baculoviruses obtained from bacmid-transfected Sf9 insect cells were used to infect suspension High Five cells (Invitrogen). Two days post-infection, the proteins were purified by binding to HITrap ProteinG HP 1 mL column (Cytiva) and eluted with 0.1M glycine-HCl and immediately neutralized by 1M Tris, pH 9.0, using an ÄKTA fast protein liquid chromatography system (GE Healthcare Life Sciences). Eluted fractions were dialyzed in PBS and concentrated using 30 KDa Amicon filter tubes (EMD Millipore). Protein concentration was determined by Qubit (Invitrogen) and stored at −80°C in aliquots.

pMslc expressing the *human Slc35A1* gene was a gift from Dr. Christopher Buck (Addgene plasmid #32095). Plasmid stock was transformed into competent DH10B cells, blasticidin selected, and plasmid DNA extracted using the E.Z.N.A. Plasmid DNA Mini Kit I (Omega BIO-TEK).

### Receptor expression

Chimeric receptors were transiently expressed in HEK *Slc35A1* KO cells by transfection of plasmids with the Transit-X2 dynamic delivery system (Mirus Bio) 24 h prior to virus (Ca’09) infection or HA-probe binding assay. A549 *Slc35A1 KO* cells stably expressing the receptor constructs were generated by transduction with pLVX-fTfR-IRES-puro, pLVX-fTfR-5J8-IRES-puro, or pLVX-Slc35A1-IRES-Neo-encoding lentiviruses in the presence of 8 µg/mL polybrene. At 48 h post-transduction, cells were subjected to selection with 1 µg/mL puromycin or 1 mg/mL neomycin.

### Analysis of receptor expression via qPCR

RNA was extracted from the indicated A549 cell lines using the ReliaPrep RNA Miniprep kit (Promega) and then reverse-transcribed into cDNA using oligo (DT) primers and Superscript IV RT (Promega) as per manufacturer’s instructions. Quantitative reverse transcription PCR (RT-qPCR) was performed with PowerTrack SYBR Green (Thermo Fisher Scientific) on a 7300 real-time PCR system (Applied Biosystems). The following primers targeting 5J8 and the extracellular domain of fTfR were utilized: fTfR fwd TGGCTGTATTCTGCTCGTGG, fTfR rev GCACTGATGTTTTCCTGGCG, 5J8 fwd GAAGGGGCTGGAGTGGATTG, 5J8 rev ATGCAGTATCGGGGTAACCA. GAPDH was included as an internal control. Samples where no amplification was detected were assigned a Ct of 40. Receptor expression for cell line validation was depicted as the 40-∆C_T_ method, where the average C_T_ of GAPDH was subtracted from the average C_T_ of the target gene. Baseline expression was calculated by assuming a C_T_ of 40 for a target gene and performing the same calculation. Relative receptor expression stability was also calculated using the 40-ΔC_T_ method as described above. Each experiment was performed in technical triplicate.

### Binding analysis with HA-probe and A/Netherlands/602/09 IAV

To assess binding of the HA-probe to the various receptors. HEK wild-type (WT), fTfR-5J8, or full-length fTfR-expressing HEK *Slc35A1* KO cells were fixed, blocked with Carbo-Free Blocking Solution for 30 min (Vector Laboratories), and incubated with the HA-hFc probe for 1 h at room temperature (RT) diluted in blocking solution. Bound HA-hFc was detected with anti-human IgG Fc Alexa 488 (Jackson Labs) and quantified using a Millipore Guava EasyCyte Plus flow cytometer (EMD Millipore), and data were analyzed using the FlowJo software (TreeStar). Microscopic data were obtained by identical staining with DAPI of receptor-expressing or HEK WT cells in a 12-well plate (pre-treated with poly D Lysine [Invitrogen]), and images were taken using a Zeiss AxioSkop HBO 50 fluorescence microscope.

To assess IAV binding to the fTfR-5J8, A549 cells stably expressing *Slc35A1*, 5J8-fTfR, or fTfR alone were inoculated with A/Netherlands/602/09 (A/Neth/09) at a multiplicity of infection (MOI) of 25 for 1.5 h on ice. Following two washes with PBS, cells were stained with LIVE/DEAD Fixable Near-IR Dead Cell Stain (Thermo Fisher Scientific) as per manufacturer’s instructions. Thereafter, cells were fixed and incubated with anti-HA 30D1 antibody (43) in FACS buffer. Bound virions in live cells were detected with anti-m-Alexa Fluor 647 (Thermo Fisher Scientific), and the signal intensities were quantified using a Becton Dickinson FACSVerse flow cytometer. Flow cytometry data were processed with FlowJo v.10.8.

### Cell infection and quantification

#### A/California/04/2009

HEK WT or HEK *Slc35A1* KO cells transiently expressing fTfR-5J8 or fTfR or the SLC35A1 were washed and incubated with the virus at an MOI of 0.2 for 1 h at 37°C. Growth media was added to each well, and infection was allowed to proceed for another 7 h. Cells were fixed with 4% PFA for 10 min, and then incubated with in-house mouse anti-nucleoprotein (NP) antibody polysera in permeabilization buffer for 1 h, then washed with 1× PBS and further stained with anti-mouse Ig Alexa 488 for another 1 h. DAPI stain was used to visualize cell nuclei. Imaging was done via the Zeiss AxioSkop HBO 50 fluorescence microscope, and images were analyzed to obtain relative infected cell counts by ImageJ (NIH) (44).

#### Neth/09-*Renilla* virus

A549 *Slc35A1* KO cells stably expressing SLC35A1 or the receptors were inoculated with Neth/09-*Renilla* virus as reported in reference 35. Cells seeded in 96-well plates were washed once with PBS and inoculated with Neth/09-*Renilla* virus at an MOI of 3 for 1 h at 37°C. Following inoculum removal, cells were washed once with PBS and maintained in DMEM supplemented with 0.1% fetal bovine serum (FBS), 0.3% BSA, 20 nM HEPES, 1% P/S (post-infection DMEM), and 6 µM *Renilla* luciferase substrate (EnduRen, Promega). The mean relative light units (RLUs) for each time point were plotted, and the area under the curve (AUC) was analyzed.

#### Endocytic entry

To assess the route of IAV entry, A549 LV-S*lc35A1* or LV-fTfR-5J8 cells were pre-treated with dynasore (40 µM–20 µM, Selleckchem), pitstop 2 (15 µM–10 µM, Sigma-Aldrich), NH_4_Cl (25 mM), or dimethylsulfoxide (DMSO) (0.15%) in DMEM for 30 min at 37°C and then inoculated with Neth/09-*Renilla* virus, vesicular stomatitis virus encoding green fluorescent protein (VSV-GFP), or respiratory syncytial virus encoding green fluorescent protein (RSV-GFP) at an MOI of 3 for 1 h at 37°C in the presence of the inhibitors. VSV-GFP and RSV-GFP were gifts from Ben Hale, Institute of Medical Virology, University of Zurich. For infections with Neth/09-*Renilla* virus, cells were maintained in post-infection DMEM supplemented with NH_4_Cl (25 mM) and 6 µM *Renilla* luciferase substrate. Luminescence measurements were acquired at the indicated times post-infection with a Perkin Elmer plate reader. For infections with VSV-GFP or RSV-GFP, cells were maintained in post-infection DMEM supplemented with NH_4_Cl (25 mM) and imaged with an Incucyte S3 Live-Cell Analysis Instrument (Sartorius). To assess GFP expression, the total integrated green object intensity (green calibrated unit × µm^2^/image) was measured and normalized to cell confluence. Mock-infected cells were utilized to determine background luminescence or GFP expression.

To corroborate viral fusion with the endosomal membrane, A549 LV-S*lc35A1* or LV-fTfR-5J8 cells were pre-treated with bafilomycin (10 nM, Sigma-Aldrich) or DMSO (0.1%) in DMEM for 2 h at 37°C and then inoculated with Neth/09-*Renilla* virus at an MOI of 3 for 1 h at 37°C in their presence. Following inoculum removal, the cells were maintained post-infection with DMEM supplemented with NH_4_Cl (25 mM) and 6 µM *Renilla* luciferase substrate, and luminescence measurements were acquired as described above.

#### Cell viability

The indicated cell lines were treated with inhibitors (dynasore, pitstop 2, or bafilomycin) or controls (NH_4_Cl or DMSO) as described above. Cells treated with dynasore or pitstop 2 were then washed once with PBS and maintained in post-infection DMEM supplemented with NH_4_Cl (25 mM). Cell viability was determined at 3 h (bafilomycin) or 24 h (dynasore, pitstop 2) post-inhibitor treatment with the CellTiter-Glo Luminescent Cell Viability Assay (Promega) according to manufacturer’s instructions. Luminescence measurements were acquired with a Perkin Elmer plate reader

### Binding and infection analysis on mutant fTfR-5J8 receptors

To evaluate the effect of receptor-HA affinity on viral entry and infection, mutant fTfR-5J8 chimeras expressing the Flag sequence (DYKDDDDK) at the N-terminus of the vH chain (Fig. 5B) were prepared by site-directed mutagenesis (GenScript).

Receptor binding to the HA-hFc probe was carried out by transfecting 0.5 µg of each plasmid DNA into HEK *Slc35A1* KO cells in a 24-well plate (Greiner Bio-One) using the Transit-X2 transfection reagent (Mirus Bio) according to manufacturer’s instructions. After 24 h, cells were detached using Accutase (Gibco), and 200 µL of the cell suspension was fixed, washed with ice-cold FACS buffer, and stained with mouse anti-Flag M2 (Sigma-Aldrich) and the HA-hFc probe for 40 min. Following two to three washes, cells were incubated with anti-mouse Alexa Fluor 488 (Invitrogen) and anti-human Fc Alexa Fluor 647 (Invitrogen) secondary antibodies for 40 min. After an additional two to three washes, HA binding was quantified.

For the infection assay, HEK *Slc35A1* KO cells transiently expressing selected mutant receptors were infected with Ca’09 at 0.5 MOI for 8 h. Following infection, cells were detached with Accutase (Gibco), and 200 µL of each cell suspension was fixed, washed with FACs buffer, and stained similar to methods for Ca’09 infection described above. Binding and infection were quantified using the BD LSRFortessa X-20 flow cytometer (BD Biosciences) with BD FACSDIVA Software (v.9.0), analyzing >10,000 cells per well. Data were processed using FlowJo v.10.9.0.

### Statistical analyses

All experiments were run in three independent replicates, except otherwise stated, and data were analyzed using GraphPad Prism (v.9.3 or 10.0) for Windows, GraphPad Software, Boston, Massachusetts, USA. Details about the specific statistical test used and data representation are described in the corresponding figure legends.

## RESULTS

### Construction and expression of an antibody-based IAV receptor

We combined anti-HA antibody heavy- and light-chain binding domains from Fab 5J8 and the cytoplasmic tail, transmembrane, and stalk sequences of fTfR to generate the artificial IAV receptor, fTfR-5J8 (Fig. 1A through E).

**Fig 2:**
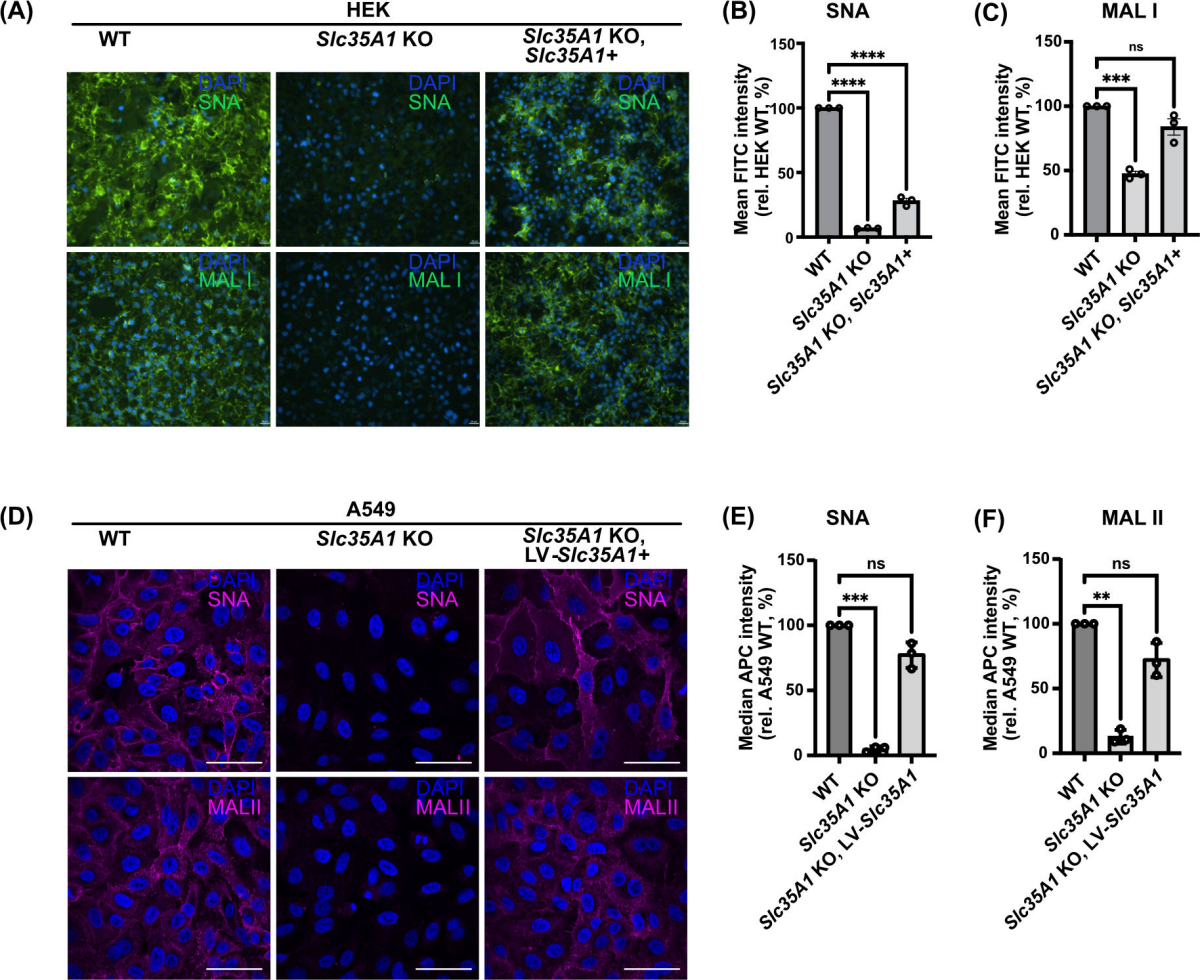
Validating Sia expression in HEK293 and A549 WT and *Slc35A1* KO conditions. (**A**) Representative unmodified fluorescent microscopy images of fixed cells stained with DAPI (blue) and fluorescein-labeled SNA or MAL I (green). Scale bar represents 20 µm. Relative fluorescence level of (**B**) SNA and (**C**) MAL I stained live cells quantified by flow cytometry with more than 10,000 cells (events) analyzed for each stained condition in *n* = 3 biological experiments. Mean fluorescence intensities (MFIs) were normalized relative to HEK WT Sia expression (**B, C**). Background (unstained) subtracted MFI values were analyzed using PRISM software. Error bars show mean ± standard mean error. Statistics were calculated using two-way analysis of variance with Tukey’s multiple comparisons test. *P* < 0.05; ****P < 0.0001, ***P < 0.001. (**D**) Unmodified (WT) and *Slc35A1* KO A549 cells transduced with an *Slc35A1* encoding lentivirus (LV-*Slc35A1*) were fixed and stained with biotinylated SNA or MAL II (magenta) and DAPI (blue). The Alexa Fluor 647-streptavidin signal intensities were assessed via microscopy. Images are representative of *n* = 3 independent replicates. Scale bar represents 50 µm. (**E, F**) Cells from (**D**) were incubated with biotinylated SNA or MAL II and the APC-streptavidin in live cells analyzed via flow cytometry. Following subtraction of the median APC signal of corresponding background samples, the median APC signal was normalized to that of WT cells. Data are means ± standard error of mean/standard deviation from *n* = 3 independent experiments. Statistical significance was inferred by two-tailed one-sample *t*-test with a theoretical mean of 100. ***P < 0.001, **P < 0.01.

To test the expression and functionality of the chimeric receptor in the absence of Sia, we expressed the chimera in an *Slc35A1* gene knockout background in two human cell lines: HEK 293 (HEK) and A549 cells. Both cell lines are permissive to IAV entry and productive infection (45, 46). Sia expression levels in *Slc35A1* KO HEK or A549 cells were confirmed by staining with SNA or MAL I/II lectins via immunocytochemistry (Fig. 2A , D) and flow cytometry (Fig. 2B and C, E, and F). SNA recognizes α2,6-linked Sia residues whereas MAL I/II binds to α2,3-linked Sias. HEK and A549 WT cells as well as the *Slc35A1*-reconstituted cells displayed higher levels of either α2,6- or α2,3-linked Sias compared to their *Slc35A1* KO versions. The KO cells exhibited a 95%–60% decrease in α2,6- and α2,3- Sia levels (Fig. 2B and C, E, and F).

### HA-hFc probe and IAV bind to fTfR-5J8-expressing cells

**Fig 3:**
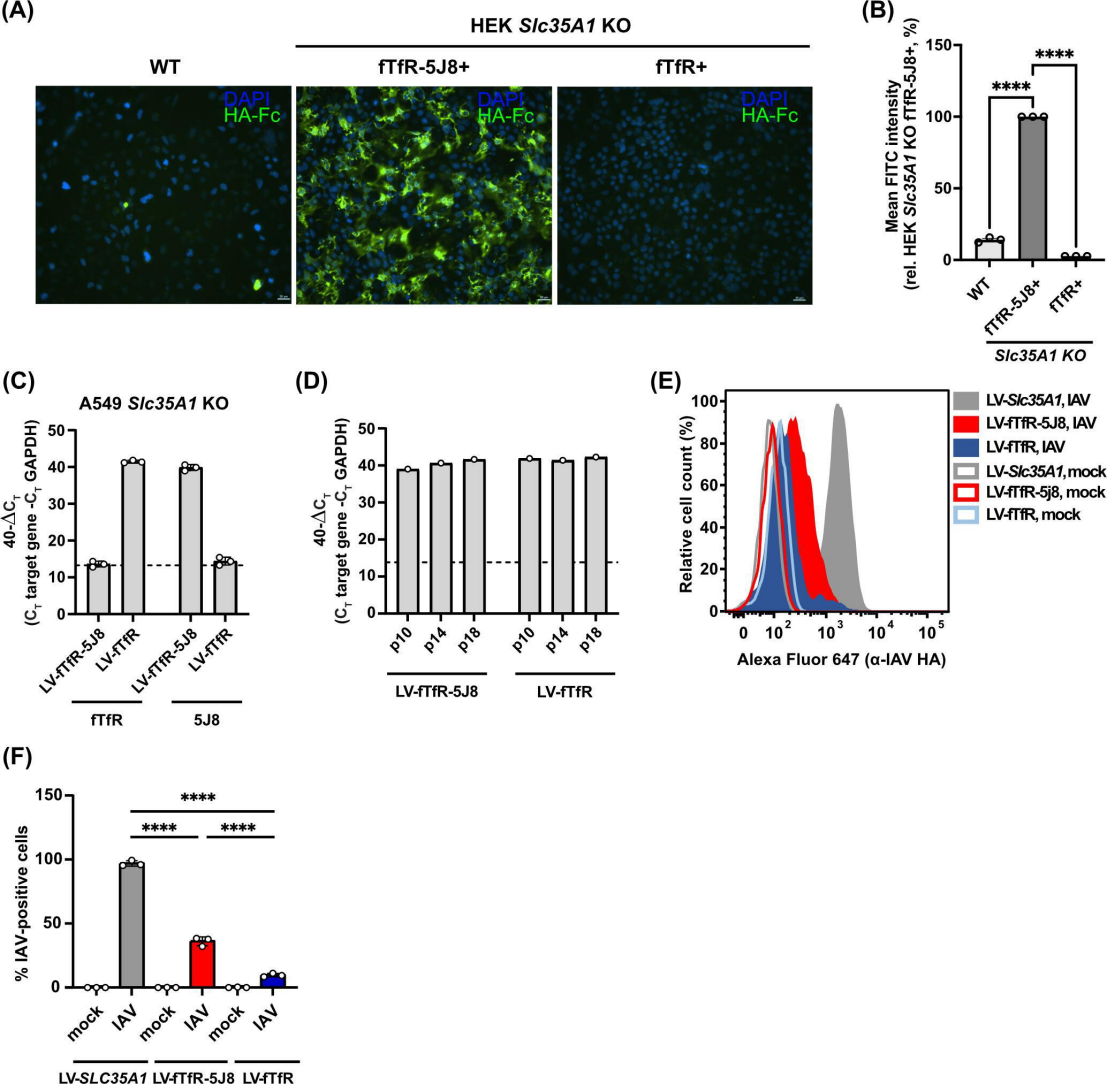
Binding analysis of HA-probe or live virus to antibody-based receptor-expressing cells. (**A**) Representative unmodified fluorescent microscopic images of fixed cells incubated with HA-hFc probe and detected with fluorescein-labeled anti-human Fc and DAPI (blue). Scale bar represents 20 µm. (**B**) Relative fluorescent intensities of live HA-hFc-stained cells were obtained via flow cytometry, with more than 10,000 cells (events) analyzed for each stained condition from *n* = 3 biological experiments. Fluorescent intensities were normalized relative to fTfR-5J8-expressing cells. Error bars show mean ± standard mean error. Statistics were calculated using two-way analysis of variance (ANOVA) with Tukey’s multiple comparisons test. *P* < 0.05; ****P < 0.0001. (**C**) The fTfR and fTfR-5J8 expression was analyzed in A549 *Slc35A1 KO* cells transduced with lentiviruses encoding fTfR (LV-fTfR) or fTfR-5J8 (LV-fTfR-5J8) via RT-qPCR. Primers targeting GAPDH, the extracellular domain of fTfR and 5J8, were utilized. Data are 40-∆C_T_ values from *n* = 3 independent experiments. A C_T_ of 40 was assigned to samples where no amplification was detected. (**D**) The receptor expression stability in cells from (**C**) was analyzed via RT-qPCR by evaluating RNA collected at passage numbers 10, 14, and 18 (p10, p14, and p18, respectively). (**E**) Cells from (**C**) and A549 *Slc35A1* KO cells stably expressing SLC35A1 (LV-*Slc35A1*) were inoculated with A/Netherlands/602/09 at an MOI of 25 for 1.5 h on ice. Cells were incubated with an anti-HA antibody, and the signal from bound virus was quantified via flow cytometry. A representative histogram from *n* = 3 independent experiments is shown. (**F**) Quantification of the percentage of IAV positive cells from (**E**). The gating strategy was established using the mock-infected sample. Data are means ± standard deviation, and statistical significance was inferred by one-way ANOVA with Sidak’s multiple comparisons test. ****P < 0.0001.

Surface expression and HA recognition of the fTfR-5J8 receptor were tested by transiently expressing fTfR-5J8 in HEK *Slc35A1* KO cells for 24 h, then incubating with an HA-hFc probe comprised of Ca’09 HA fused to a human IgG1 Fc domain. Flow cytometry analysis of the stained non-permeabilized cells showed ~100-fold increased fluorescence intensity compared to control cells expressing the full-length fTfR (Fig. 3A and B). The HEK WT cells also bound the HA-hFc probe, albeit to a much lower level (Fig. 3B). Next, we evaluated the binding of A/Netherlands/602/09 virions (99% HA sequence identity to Ca’09) to A549 *Slc35A1* KO cells stably expressing either fTfR-5J8, SLC35A1, or full-length fTfR (Fig. 3C). Receptor expression levels appear not to be negatively affected upon multiple rounds of passages (Fig. 3D). Virions showed significantly higher binding to cells expressing the fTfR-5J8 receptor compared to those expressing the full-length fTfR control (Fig. 3E and F), thus confirming the expression of the fTfR-5J8 receptor on the cell surface and its recognition of the pH1N1 HA antigen. *Slc35A1*-reconstituted cells showed significantly greater virus binding, as expected (Fig. 3E and F).

### Antibody-based receptor rescues viral infection in Sia-deficient cells

**Fig 4:**
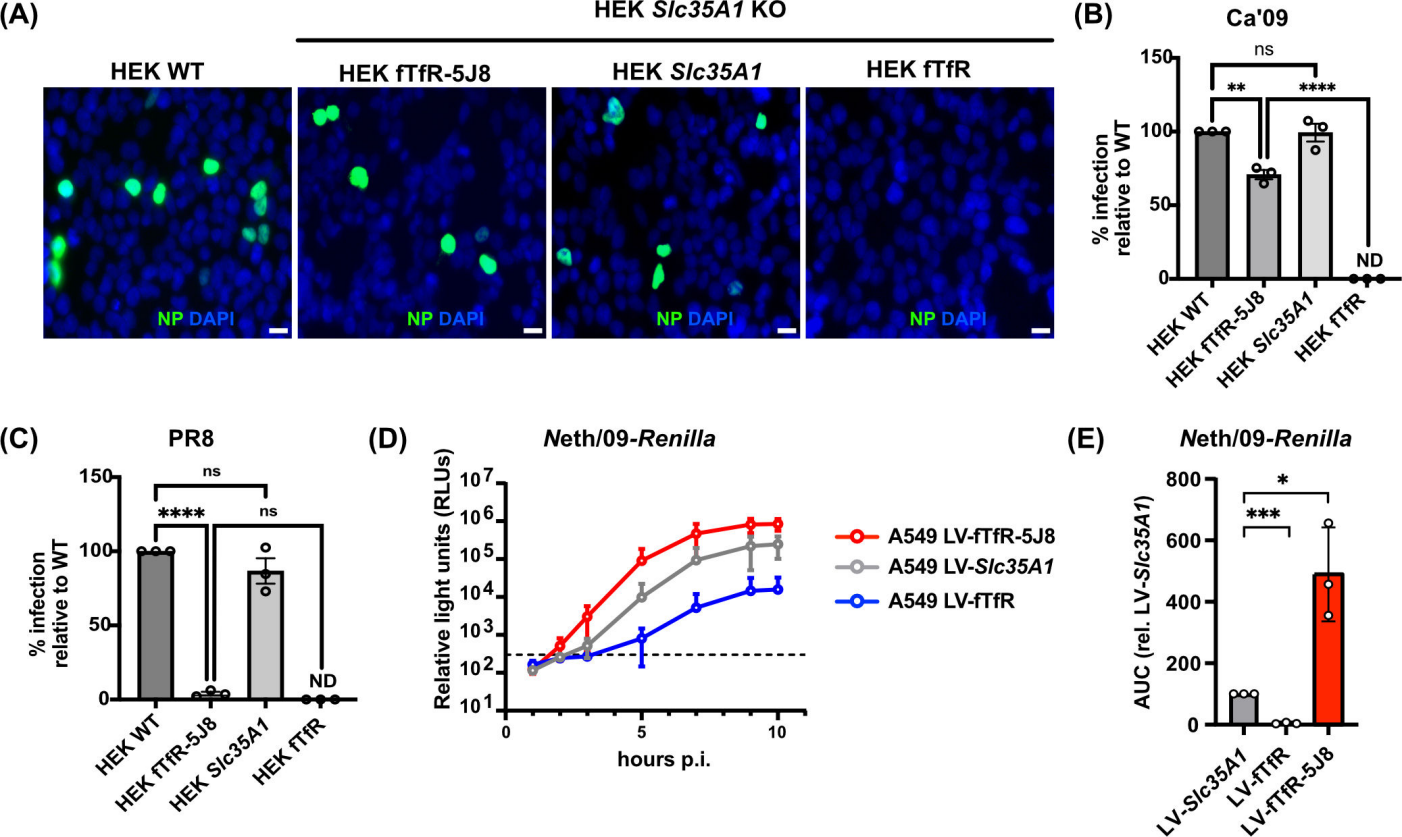
Antibody-based receptor rescues IAV infection in the absence of Sia. (**A**) Representative fluorescence microscopy images of fixed Ca’09-infected cells. Receptors (fTfR-5J8 and full-length fTfR) were transiently expressed on HEK *Slc35A1 KO* for 24 h before viral infection (MOI = 0.2) for 8 h. Cells were fixed and stained with anti-NP antibody (green) and DAPI (blue). (**B, C**) Infection data for Ca’09 or PR8 infected cells, respectively. Four different fields of view were imaged for each condition, and percent infection was determined. Data were normalized to HEK WT infected cells. (**D**) Infection curve for the Neth/09*-Renilla* at MOI = 3 in A549 LV-fTfR, LV-fTfR-5J8, and LV-*Slc35A1* cells. (**E**) AUC plot from D, where AUC values of LV-fTfR-5J8 and LV-fTfR are shown relative to LV-*Slc35A1*. ND, no infection detected; NS, not significant. All experiments were performed in three independent replicates (*n* = 3). Error bars show mean ± standard mean error. Statistics were calculated using two-way analysis of variance with Tukey’s multiple comparisons test. *P < 0.05; **P < 0.01, ****P < 0.0001.

To test whether IAV can infect cells after binding to the fTfR-5J8 receptor, we inoculated the fTfR-5J8-expressing or control HEK cells with virus at a multiplicity of infection of 0.2 and incubated the cells for 8 h (Fig. 4A), which corresponds to one full replication cycle (47). Results are shown as fluorescent antibody staining for the NP (Fig. 4A and B) and the percentage of cells infected, which was normalized to the percentage seen for the WT control cells. The fTfR-5J8 receptor rescued infection to ~70% of Ca’09 WT infection in the *Slc35A1* KO HEK cells (Fig. 4B). Cells transiently reconstituted by expression of *Slc35A1* from a plasmid displayed similar levels of IAV infection to HEK WT cells, while no Ca’09 infection was detected in the control fTfR-expressing cells (Fig. 4B). To assess the specificity of the fTfR-5J8 receptor, we asked whether it could rescue H1N1 strains other than those of pandemic origin. We tested this using the laboratory-adapted H1N1 strain A/PR/8/1934 (PR8) strain, which was previously shown not to bind to 5J8 IgG or Fab (36). As expected, our results show that the fTfR-5J8 receptor did not rescue infection of PR8 HEK *Slc35A1* KO cells (Fig. 4C).

We further examined the functionality of the artificial receptor by infecting the *Slc35A1* KO A549 cells transduced to express fTfR-5J8, fTfR, or SLC35A1 with the pH1N1 A/Netherlands/602/2009 strain expressing the *Renilla* luciferase gene (Neth/09-*Renilla*) (35). Live cell luminescence readout of the various A549 cells inoculated with Neth/09-*Renilla* over the course of 10 h at an MOI of 3 showed results similar to that of the Ca’09 infection (Fig. 4D). RLU and AUC plots of the infected fTfR-5J8-expressing cells were ~80- to 90-fold greater than those of the control fTfR-expressing cells and were also enhanced relative to *Slc35A1-*reconstituted LV-transduced A549 cells (Fig. 4D and E).

### Reduced affinity of antibody-based receptor for HA antigen enhances infection

**Fig 5:**
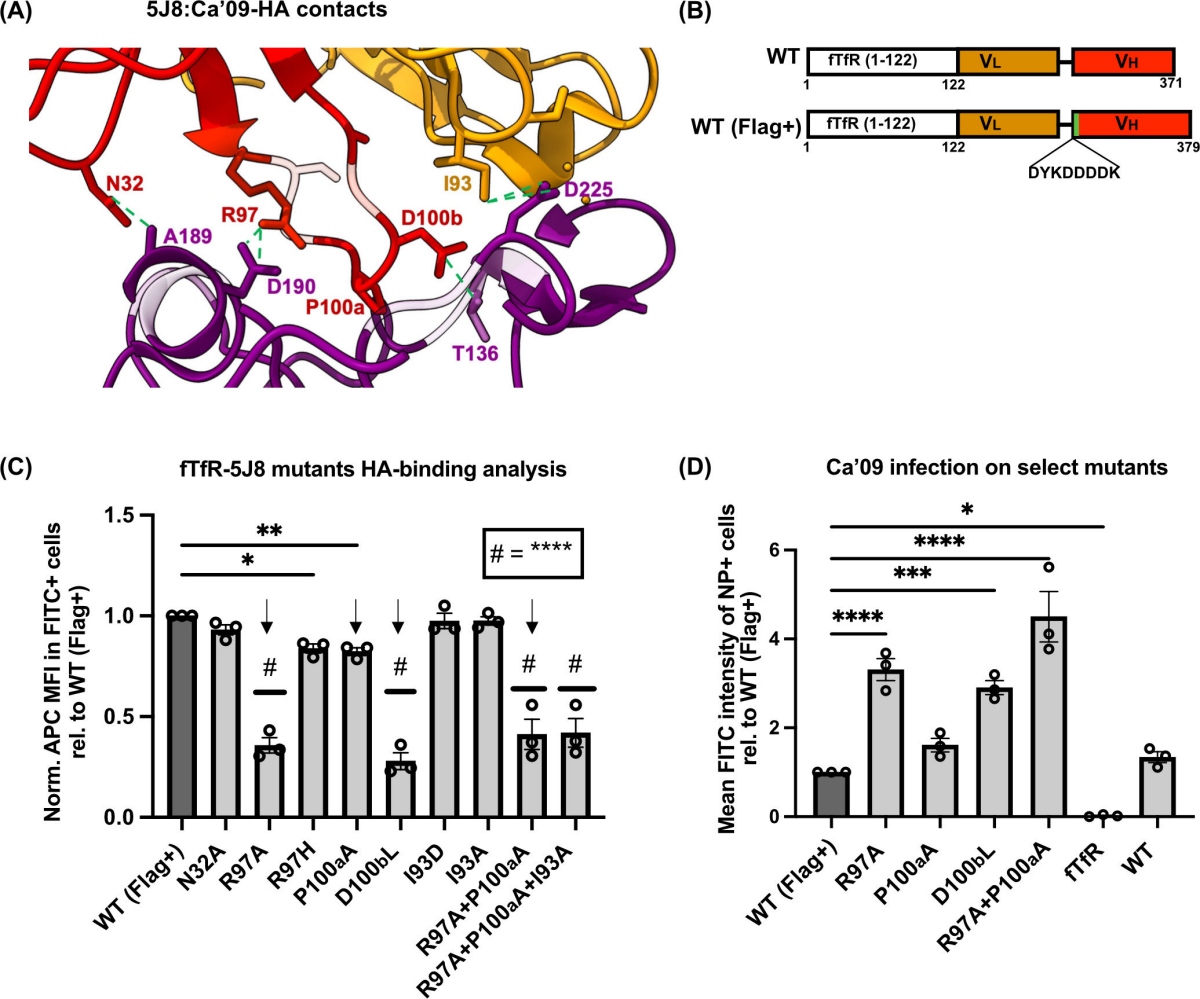
Receptor mutants binding and infection analysis. (**A**) Fab 5J8 and A/Ca/07/2009 HA complex (PDB: 4M5Z) showing interacting residues between antibody complementarity-determining region (CDRs) and the HA antigen. Intermolecular contacts were visualized using Chimera X (53) (green dashed lines). Red (HCDR3 loop), gold (LCDR3), and purple (receptor binding site [RBS] pocket, HA antigen). (**B**) Cartoon showing the fTfR-5J8 construct (WT) and the Flag tag version (WT [Flag+]). Mutagenesis was carried out using the WT (Flag+) construct. (**C**) Receptor: HA-hFc binding analysis data. Fixed HEK *Slc35A1* KO cells transiently expressing receptors were incubated with an anti-Flag antibody and the HA-hFc probe. Fluorescent signals were detected using flow cytometry on the FITC (anti-Flag) and APC (HA-hFc) channels. Binding strength to the HA-hFc probe was quantified as the relative mean fluorescence intensity (MFI) of APC in Flag+ cells, normalized to the mean FITC signal. The Flag+ population was gated using cells expressing only fTfR or original WT receptor. (**D**) Selected receptor mutants transiently expressed in HEK *Slc35A1* KO cells were infected with the Ca’09 virus at an MOI of 0.5 for 8 h. Fixed cells were permeabilized and stained with an anti-NP antibody to detect infected (NP+) cells via flow cytometry on the FITC channel. Mean FITC intensity was expressed relative to the WT (Flag+) control. Final MFI values were calculated by subtracting the signal intensity of mock-infected cells. All experiments were performed in three independent replicates (*n* = 3). Error bars show mean ± standard mean error. Statistics were calculated using two-way analysis of variance with Tukey’s multiple comparisons test. *P < 0.05; **P < 0.01; ***P < 0.001; ****P < 0.0001. Only significant values are indicated in the graphs.

The affinity of cellular receptors for viral antigens has been shown to influence viral entry and infection (48–52). To investigate whether altering the binding strength of the fTfR-5J8-HA domain affects viral rescue and infection in the absence of sialic acid, we introduced mutations in key residues of the HA-binding region of the fTfR-5J8 receptor. Based on the structure of the Fab 5J8:Ca’09 HA complex, five critical residues including the D100b on the HCDR3 loop (important for Sia mimicry) were targeted (Fig. 5A). A total of nine mutant constructs were generated on a Flag-tagged version of the fTfR-5J8 receptor (WT [Flag+]) (Fig. 5B and C).

HA-binding analysis using the HA-hFc probe on HEK *Slc35A1* KO cells transiently expressing these mutant receptors showed that six constructs exhibited significantly reduced HA binding (Fig. 5C). Among these, the R97A, P100A, and the double mutant R97A + P100A demonstrated more than 50% reduction in binding compared to the WT receptor. Subsequent infection experiments using 0.5 MOI of Ca’09 virus for 8 h on HEK *Slc35A1* KO cells expressing four of the six mutants with reduced HA-binding levels revealed significantly increased viral infection. Flow cytometry analysis of NP-positive cells (Fig. 5D) showed that the R97A, D100bL, and R97A + P100A double mutant had more than a 2.5-fold increase in infection compared to the WT receptor. These findings indicate that reduced receptor binding strength for the virus may enhance viral uptake and infection.

### Antibody-based receptor uptake employs a similar endocytosis pathway as Sia-mediated entry

**Fig 6:**
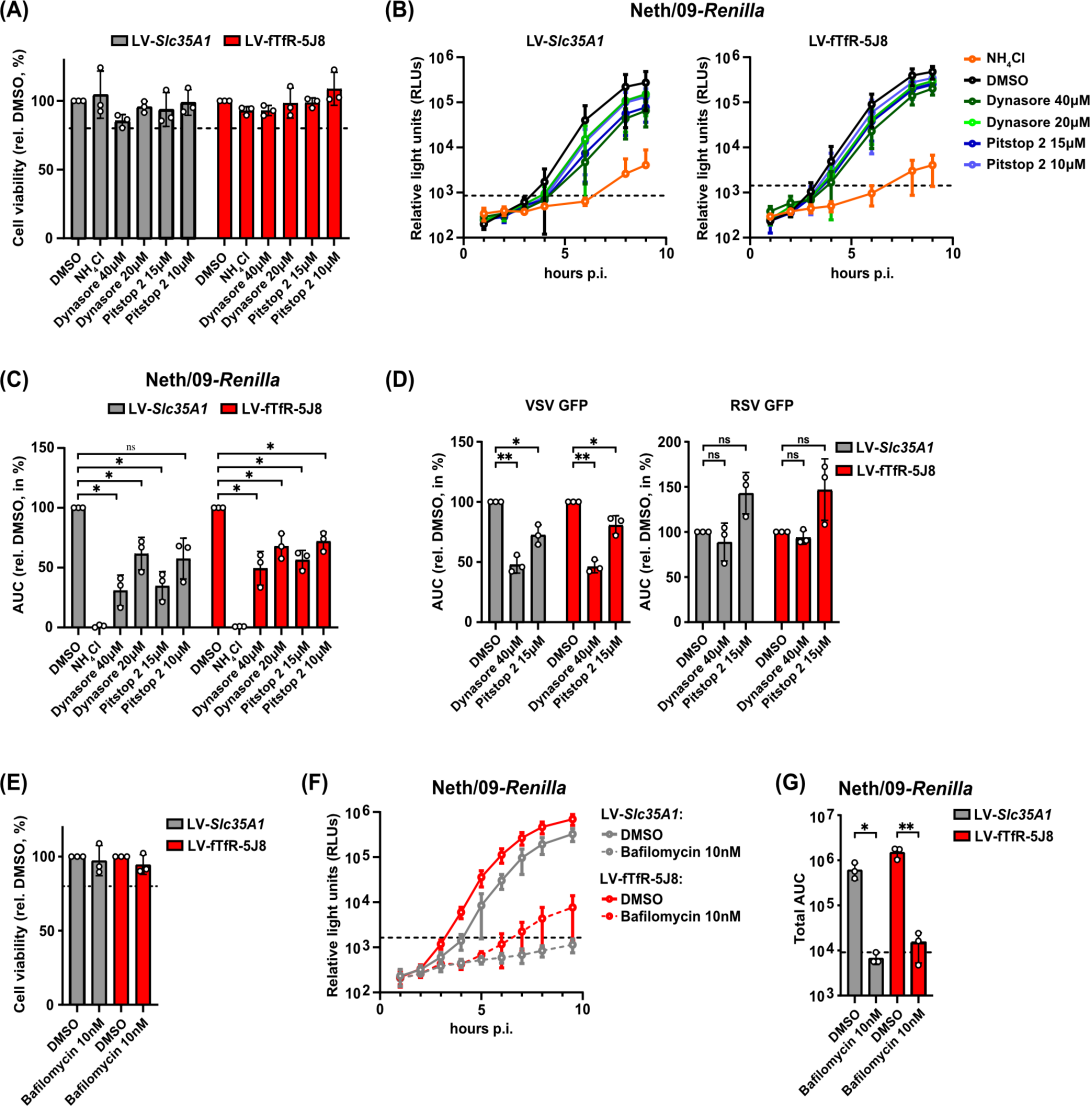
IAV entry mediated by the antibody-based receptor is dynamin- and clathrin-dependent. A549 *Slc35A1* KO cells stably expressing SLC35A1 (LV-*Slc35A1*) or fTfR-5J8 (LV-5J8-TfR) were pre-treated with dynasore (40 µM–20 µM), pitstop 2 (15 µM–10 µM), NH_4_Cl (25 mM), or DMSO (0.15%) for 30 min at 37°C and then infected with the indicated viruses at an MOI of 3 in their presence. (**A**) Cell viability at 24 h post-inhibitor treatment relative to the DMSO control. (**B**) Neth/09*-Renilla* infection curves. (**C**) AUC values from (**B**) until 9 h post-infection were normalized to the DMSO-treated sample within each cell line. (**D**) AUC values for VSV-GFP and RSV-GFP infection curves. AUC values for VSV-GFP or RSV-GFP infections were calculated until 11 or 27 h post-infection, respectively, and were normalized to the corresponding DMSO-treated samples. (**E–G**) A549 LV-*Slc35A1* and LV-5J8-fTfR cells were pre-treated with bafilomycin (10 nM) or DMSO (0.1%) for 2 h at 37°C and inoculated with Neth/09-*Renilla* as in (**A**). (**E**) Cell viability at 3 h post-inhibitor treatment relative to the DMSO control. (**F, G**) Neth/09*-Renilla* infection curves (**F**) and the corresponding total AUC values until 9 h post-infection (**G**). (**A–F**) Data are means ± standard deviation from *n* = 3 independent experiments. Statistical significance was determined by two-tailed one-sample *t*-test with a theoretical mean of 100 (**C, D**) or by two-tailed unpaired *t*-test. *P < 0.05, **P < 0.01; ns, not significant. The dashed line indicates 80% cell viability (**A, E**), the luminescence from the mock-infected sample (**B, F**) or the AUC for the mock-infected sample (**G**).

As a major IAV entry route in the absence of serum is CME**,** we next investigated whether this was the case in the antibody-based receptor system. A549 *Slc35A1* KO LV-*Slc35A1* and LV-fTfR-5J8 cells were treated with inhibitors targeting dynamin (dynasore) or CME (pitstop 2) and then infected with A/Neth/09-*Renilla* virus in their presence. Inhibitor treatment did not impact cell viability (Fig. 6A) and led to a dose-dependent decrease in viral replication in cells with reconstituted Sia expression and those overexpressing the antibody-based receptor (Fig. 6B and C). As both inhibitors have been reported to have non-specific effects (54, 55), we also evaluated their role in the replication of VSV-GFP and RSV-GFP, which enter cells through CME and fusion at the cell surface or macropinocytosis, respectively (56–58). Inhibitor treatment led to decreased replication of VSV-GFP, but not RSV-GFP, in both cell lines (Fig. 6D). Moreover, treatment with bafilomycin, an endosomal acidification inhibitor, significantly reduced A/Neth/09-*Renilla* virus replication at non-cytotoxic concentrations (Fig. 6E through G). In summary, IAV entry mediated by the fTfR-5J8 receptor is dependent on dynamin, CME, and endosomal acidification, akin to that in a Sia-positive context.

## DISCUSSION

Here, we have developed a new tool for the study of influenza virus binding, entry, and infection of cells by preparing a glycoprotein receptor that binds specifically to the same region of HA as Sia receptors. By using a protein ligand that combines an antibody as a defined binding structure and a truncated receptor with a well-understood cell entry pathway involving clathrin-mediated endocytosis, we can use the fTfR-5J8 receptor to both understand and manipulate the processes involved. For multiple IAVs, studies over the past 70 years have shown that viral infection requires Sia binding. It is also clear that differences in the affinity of Sia binding are directly correlated with the efficiency of infection, for example, those seen between the α2,3- and α2,6-Sia linkages found in birds and humans, respectively (59, 60). However, we still lack a clear understanding of how that binding leads to uptake or entry and infection (61). It is difficult to modify Sia receptors on cells or in animals, as the Sia, oligosaccharides, and glycoproteins or glycolipids that form the functional receptors are the products of diverse and variable series of enzymatic activities, making it difficult to change the receptor properties with any precision or predictability.

Previous studies involving chimeric systems to study virus internalization into non-permissive environments have shown mixed results (37, 62–64). For instance, scFv-fTfR chimeras designed for canine parvovirus uptake resulted in internalized virions without achieving infection (37). Conversely, scFv-ICAM1 chimera for foot-and-mouth disease virus (FMDV) resulted in successful uptake and infection into non-FMDV-susceptible cells (63). This suggests that mere recognition by the chimeric viral-binding domain may be insufficient for successful uptake and infection of some viruses.

**Fig 7:**
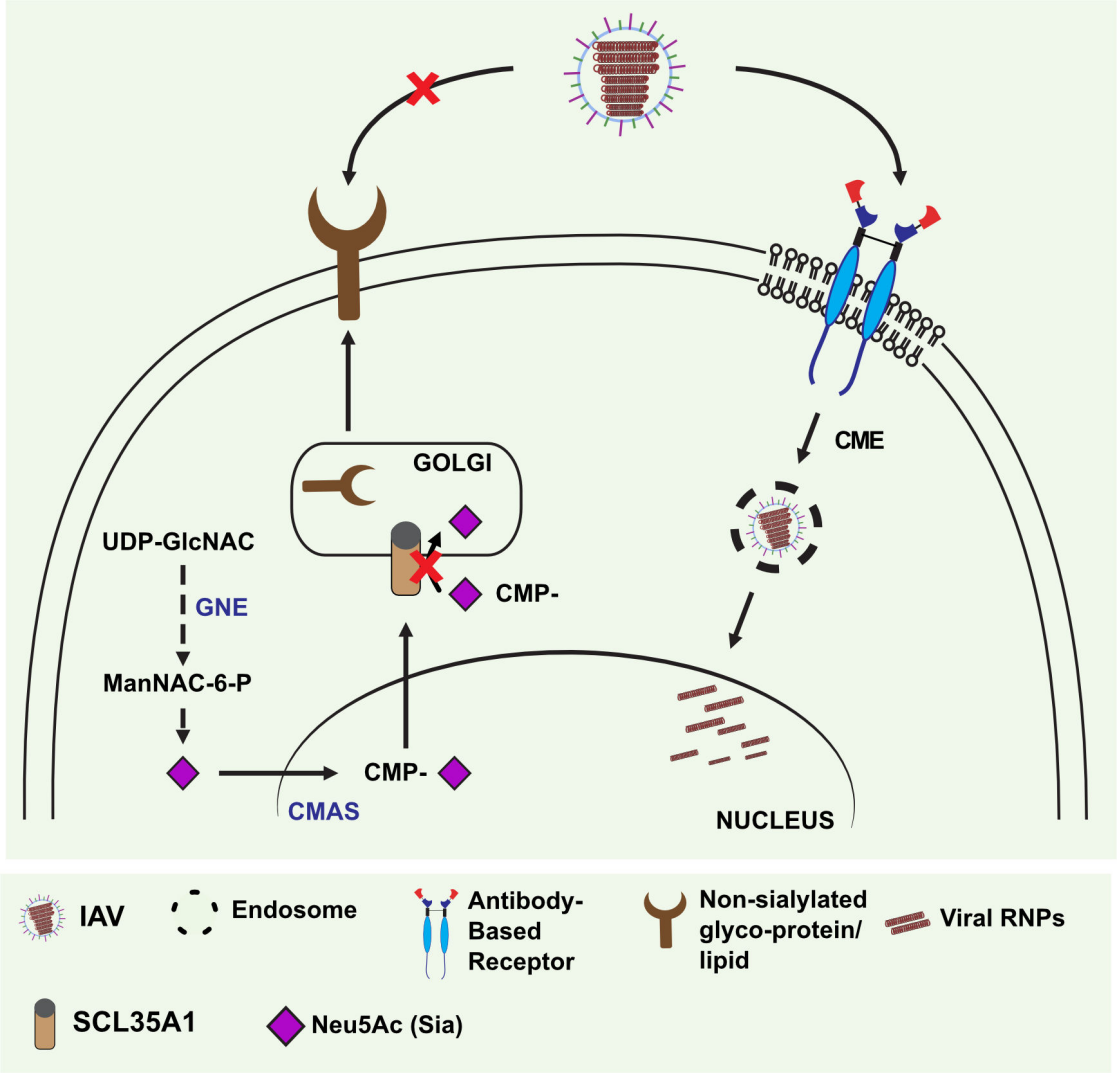
Summary of the new receptor prepared here and its potential for analysis of the IAV entry pathways. HEK and A549 *Slc35A1* KO cells were prepared, allowing us to examine the rescue of IAV uptake, internalization, and infection in the absence of efficient Sia surface expression by using the alternative receptor. Schematics of the Sia biosynthetic pathway are shown (summarized in reference 68), indicating the accumulation of CMP-Sia in the cytosol due to the gene deletion of the antiporter SLC35A1. UDP-GlcNAC, uridine diphosphate N-acetylglucosamine; ManNAC-6-P, N-acetyl-mannosamine 6-phosphate; GNE, UDP-GlcNAc 2-epimerase/ManNAc-6-kinase.

In this study, the fTfR-5J8 receptor combines the HA-binding domain as an scFv of an anti-pH1N1 antibody (MAb 5J8) fused to the cytoplasmic tail, transmembrane, and stalk domain of the feline transferrin receptor type-1. Uptake of the TfR and its regular ligand holo- (iron-bound) transferrin (Tf) occurs almost entirely via CME due to the efficient engagement of the TfR cytoplasmic tail with the adaptor protein 2, which links it to clathrin-coated pits (65, 66). The receptor was expressed on the cell surface where it bound to the viral HA—as shown for the HA-hFc fusion protein. It also efficiently rescued pH1N1 infection on both HEK293 and A549 cells that lack Sia cell surface expression due to knockout of the Sia-CMP transporter, SLC35A1(67) (Fig. 7). Notably, the rescue of infection was comparable to that observed in *SLC35A1*-reconstituted cells. The fTfR-5J8-mediated uptake appears to follow the CME pathway similar to Sia-expressing cells, as evidenced by its comparable sensitivity to dynasore and pitstop, inhibitors of dynamin- and clathrin-mediated endocytosis, respectively.

Our findings align with previous studies showing that the influenza virus can directly infect B cells expressing surface HA-specific B cell receptors (BCRs), where antigen recognition by the BCRs renders the B cells susceptible to infection (69). Interestingly, we observed enhanced infection rates with lower-affinity receptor mutants, suggesting that modulating antibody–HA interactions can significantly influence infection. Furthermore, our data indicate that these processes might be independent of the Sia mimicry properties of the 5J8 HA-binding domain. The D100bLeu mutation, which likely disrupts the Sia mimicry of 5J8 (36), still enhanced IAV infection. Carrying out infection rescue with a receptor that does not mimic Sia or bind the RBS will test this hypothesis further.

### Future studies

In follow-on studies, we will more closely examine the entry and infection pathways of the H1N1 pandemic viruses by manipulation of the properties of the fTfR-5J8 receptor. For example, targeted mutagenesis of key sequences within the TfR cytoplasmic tail can be used to at least partially redirect receptor trafficking into alternative endosomal pathways, potentially revealing any novel mechanisms of viral uptake and infection (37, 70). Additionally, other aspects of the viral infection process might also be revealed, including a better understanding of the role of the NA and cell surface binding in the viral entry (71–73).

This chimeric antibody-based receptor system may present opportunities for generating transgenic *in vivo* models to study influenza pathogenesis. By expressing non-canonical IAV receptors in mice or other systems, it may be possible to create models for infection with viral strains that are typically non-pathogenic in those systems due to absence of compatible receptors. This approach could provide valuable insights into virus–host interactions, immune responses, and potential therapeutic interventions.

In summary, we believe this type of receptor provides a novel and versatile platform for exploring influenza virus biology, enabling precise manipulation of entry pathways and allowing us to explore innovative model systems to define processes involved through non-canonical host–virus interactions.
